# Long-distance winter migrations of chinstrap penguins and elephant seals to a persistent bloom at the edge of the Ross Gyre

**DOI:** 10.1038/s41598-025-87433-6

**Published:** 2025-03-21

**Authors:** Cara Wilson, Jefferson T. Hinke, Matthew Mazloff

**Affiliations:** 1 Information Technology and Data Services Division, Southwest Fisheries Science Center, National Marine Fisheries Service, National Oceanic and Atmospheric Administration, Monterey, CA USA; 2 U.S. Antarctic Marine Living Resources Program, Ecosystem Science Division, Southwest Fisheries Science Center, National Marine Fisheries Service, National Oceanic and Atmospheric Administration, La Jolla, California, CA UK; 3 Scripps Institution of Oceanography, University of California, San Diego, La Jolla, California, CA UK

**Keywords:** Chinstrap penguins, Ross Gyre, Amundsen Sea, SOCCOM data, Southern elephant seals, Biogeochemistry, Ocean sciences

## Abstract

Assessing the biological characteristics of high-latitude winter habitats of migratory marine predators is necessary for conservation and management in Antarctica. Tracking data from chinstrap penguins (*Pygoscelis antarcticus*) and southern elephant seals (*Mirounga leonina*), key Antarctic predators with different diets and foraging habits, indicate that some individuals undertake long-distance winter migrations to remote regions south of 55°S and west of 120°W. There, localized hotspots of increased use, with general reductions in mean swimming speed are evident. Presumably, these predators migrate to areas with higher productivity, however the marine productivity in this remote region during winter is unknown. Light limitation during winter precludes the use of optical satellite data to characterize marine productivity here, but biogeochemical-Argo floats can provide year-round chlorophyll data. These data inform the Biogeochemical Southern Ocean State Estimate (B-SOSE), which provides year-round estimates of marine productivity. The predator hotspots overlap with two areas with year-round elevated surface chlorophyll levels predicted by B-SOSE, consistent with previous studies indicating enhanced mixing in those areas. Our results suggest that persistent areas of elevated chlorophyll centered near 160°W and 120°W near the boundaries of the Ross Gyre and the southern boundary of the Antarctic Circumpolar Current support a productive food web capable of supporting the diverse foraging niches of pelagic species during winter.

**Supplementary Information:**

The online version contains supplementary material available at 10.1038/s41598-025-87433-6.

## Introduction

Identifying and assessing the biological characteristics of the habitats used by seabirds and marine mammals during their winter migrations is an important task for understanding population trends and managing living marine resources. In particular, temporal variability in the availability and productivity of winter habitats in the Southern Ocean may help explain trends in breeding populations of Antarctic predators^1^. For example, prior work with Pygoscelid penguins suggested the importance of winter foraging conditions for explaining divergent population trends^2^. However, the marine productivity in remote Antarctic regions during winter, presumably a key attractor for migratory predators, remains unknown. Measuring marine productivity and other biological characteristics of habitats used by predators in the Southern Ocean during the austral winter is difficult because low light levels preclude traditional satellite-based observations that require visible light. Here, we use model output informed from biogeochemical-Argo float data^3^ to examine the winter distribution of marine productivity to assess and characterize a remote habitat used by migratory chinstrap penguins (*Pygoscelis antarcticus*) and southern elephant seals (*Mirounga lionina*).

Chinstrap penguins (CHPE) have a global population of roughly 8 million individuals^4^ and are primarily consumers of Antarctic krill (*Euphausia superba*) and pelagic fishes^5,6^. Southern elephant seals (SES) have a global population of roughly 325,000 individuals^7^and are primarily consumers of squids and pelagic fishes^8^. These abundant Antarctic predators account for large proportions of total krill and squid consumption in the Southern Ocean among seabirds and pinnipeds, respectively^9^. Both species routinely move long distances throughout the Southern Ocean during their winter migrations, with maximum recorded movements from breeding locations to winter destinations exceeding 4000 km^10,11^. Available tracking data from CHPE and SES record movements of several individuals, in multiple years, into a remote, high-latitude region between 55°S and 65°S and 110°W and 165°W spanning the western boundary of the Amundsen Sea and the northern Ross Sea gyre^1,11^. We will refer to this location as our focus region. There are no nearby continents to this region at the southern extent of the South Pacific, making it difficult for research vessels to get to, and it is understudied relative to other parts of the Southern Ocean^12,13^. Consequently, the ecological importance of this region for pelagic food webs is largely unknown^14^.

The interaction between the Antarctic Circumpolar Current (ACC) and the Pacific Antarctic Ridge in this region leads to enhanced mixing and nutrient upwelling^15,16^. In particular, the area around 140°W is the location of the narrowest restriction of the ACC, due to topographical steering from the underlying Udintsev Fracture Zone (UFZ) at 144°W^17^. Downstream of the UFZ, between 140°−135°W and 56°−58°S, is an area of enhanced eddy kinetic energy^17^and strengthening gradients in the circumpolar fronts^18^. Previous analyses of Southern Ocean satellite data report elevated summer chlorophyll in this region which has been attributed to the enhanced mixing caused by the interaction of the ACC and bottom topography^16,19,20^.

Characterizing marine productivity and the prey field in high-latitude habitats used by migratory animals during winter months is more difficult. The surface concentration of chlorophyll is a commonly used index of marine productivity since it can be measured by satellites. While chlorophyll is not directly consumed by marine predators, the trophic chains supporting most marine predators initiate with the phytoplankton and the grazers directly dependent on algal blooms. Thus, areas of high chlorophyll concentration are key drivers of marine productivity that fuel predators. While satellite ocean color data is often used to map surface chlorophyll concentrations, it requires the presence of both visible light and cloud-free conditions. Hence passive optical satellite sensors cannot provide chlorophyll data during Antarctic winters due to insufficient light. Thus, the high-latitude habitats of Antarctic predators during winter are usually modeled on the basis of physical habitat descriptors including sea ice coverage, sea surface temperature, depth, or proximity to frontal features of the ACC^11,21,22^. While these physical variables provide important proxies of habitat requirements, a fuller exploration of biological indices in the winter habitats used by migratory predators may help strengthen understanding of migratory behavior and, therefore, improve assessment of key overwinter habitats.

Biogeochemical-Argo (BGC-Argo) floats, deployed by the Southern Ocean Carbon and Climate Observations and Modeling program (SOCCOM; http://soccom.princeton.edu*)*, provide year-round, in situ observations of chlorophyll, as well as data from under-ice^23,24^. Despite the large number of floats deployed by SOCCOM (over 250 since 2014) the spatial coverage of float data remains relatively sparse. Thus, direct mapping of animal locations to raw float data remains untenable. However, the BGC-Argo float data has been incorporated into the Biogeochemical Southern Ocean State Estimate (B-SOSE), a general circulation model of the Southern Ocean. The B-SOSE model generates year-round estimates of biogeochemical properties for the Southern Ocean^3^, including chlorophyll concentrations, While chlorophyll is not directly consumed by top predators such as CHPE and SES, it can be used as an index to assess the general productivity of the system^25^. The spatially resolved estimates of chlorophyll during winter may help understand animal occupancy in remote high-latitude pelagic regions.

Here we use overwinter tracking data of CHPE and SES along with B-SOSE model output to examine whether variation in migratory movements in the western Pacific sector of the Southern Ocean correspond to unique features of B-SOSE predictions of ocean productivity. We also provide comparisons of B-SOSE output and satellite-derived surface chlorophyll measurements during summer to help validate B-SOSE predictions. Our results suggest that consistently elevated marine production centered near 160°W and 120°W in association with the Ross Gyre and southern boundary of the ACC may influence the long-distance winter migrations of these two iconic Southern Ocean predators.

## Materials and methods

### Telemetry data

We used publicly-available location estimates of the overwinter migrations of CHPE and SES collected from ARGOS satellite tracking data. Tracking data collected by the U.S. Antarctic Marine Living Resources Program for CHPE and reported previously^26^ were obtained from the Seabird Tracking Database, available at https://www.seabirdtracking.org. Additional CHPE tracking data were obtained from a published supplemental data file^11^ available at 10.1371/journal.pone.0226207.s00811Telemetry data for SES were obtained from the Marine Mammals Exploring the Oceans Pole to Pole (MEOP) program^27^ available at https://www.meop.net/and the Tagging of Pelagic Predators (TOPP) program^28^ available at https://coastwatch.pfeg.noaa.gov/erddap/.

The data included 97 CHPE tagged in the northern Antarctic Peninsula region in February or March of six different years between 2000 and 2017. The initial SES dataset included 1029 animals tagged all around Antarctica between 2004 and 2019. Since we are interested in the movements of SES into the Ross Gyre/Amundsen Sea, the SES dataset was subset to include only the tracks with tagging locations between 50°W and 130°E; all individuals tracked into the study region were tagged within that range. Further, because we are interested in understanding winter habitat use, where we define winter as the time from 1 April to 30 November, CHPE and SES tracks that terminated before 1 April were excluded. We also removed any tracks with fewer than 10 days of data. These criteria reduced the dataset to 74 CHPE and 315 SES tracks (Table 1).

**Table 1:** Statistics for the chinstrap penguins and southern elephant seals tags analyzed, including the total number of tags, the total locations, the average number of locations per tag, the average number of days recorded in a tag, and the average maximum distance traveled. The statistics are given for the entire dataset, and for the subset which migrated to the area centered around 140°W.

	CHPE	CHPE (in focus area)	SES (50°W-130°W)	SES (in focus area)
Total tags	74	9 (12%)	315	16 (5%)
Years	2000, 2004, 2006, 2010, 2011, 2017	2006, 2010, 2011, 2017	2004–2019	2005, 2006, 2008, 2009, 2010, 2012, 2013
Total locations	28,270	3638 (13%)	165,027	2953 (2%)
N per tag	382 ± 317	722 ± 467	524 ± 326	599 ± 214
Days per tag	94 ± 49	151 ± 53	191 ± 81	209 ± 56
Maximum distance	1085 ± 1237	3823 ± 730	960 ± 1035	3615 ± 959

Using the raw data, we assessed all 389 winter tracks to estimate maximum migration distances achieved to compare the general winter migratory behaviors of CHPE and SES. We then subset the data to isolate animals that moved into the study region. This large region was selected to encompass ice-free areas of the northern and eastern Ross Sea gyre and western Amundsen Sea. Nine CHPE and 16 SES entered the study region.

For the subset of CHPE and SES that entered the study region, we note that their satellite tags were programmed to report positions at different temporal frequencies depending on the species and year of deployment (Supplemental Table S1). While the SES tags reported data daily, some CHPE tags only reported data every 3 days. To standardize the data for analysis, a random walk model, as implemented in the ‘aniMotum’ package^29,30^for R^31^was fitted to the raw location data to predict locations along the tracks on a daily (24-hr) interval. The daily resolution of location estimates precludes analysis of fine-scale movement patterns that may be associated with, for example, individual foraging behaviors, but does enable broad-scale comparison of longer-term migratory behavior. The standardized daily locations were used to identify areas of high use during winter months with a 2-dimensional kernel density estimation (KDE) implemented in the ‘MASS’ package^32^in R^31^. The KDE was implemented independently for CHPE and SES and specified based on 100 grid points in x and y directions with the bandwidth set as the mean from two default methods^32,33^ to achieve a balance, given the tracking data, between a smoother and a more granular density surface.

Migratory predators typically transit between important breeding and wintering habitats. For marine predators that also forage during their migration between such habitats, identifying changes in behavior along their migratory path may help indicate the location of important wintering habitats. To examine whether movement behaviors changed during the tracking period, we calculated the daily speeds along each predicted track based on the shortest geodesic distance between positions^33^. We assume that periods with higher daily speeds represent transit periods, while periods with slower average speeds may indicate increased residency within important wintering habitats. Due to the predominantly east-west orientation of movement required to transit from tagging locations into the study region, we grouped the estimates of daily swim speed into 5° longitudinal bins to examine differences in swimming speed outside and inside the study region. For reference, note that a mean daily swim speed of 0.5 m/s equates to a daily displacement of 43.2 km.

## Model data

The B-SOSE model^3^ was used to examine the distribution of chlorophyll over the study area. B-SOSE is a general circulation model that assimilates observations from biogeochemical-Argo floats, shipboard data, and satellites to produce a realistic estimate of the ocean’s physical and biogeochemical states. The model output used is “Iteration 135” and is available at http://sose.ucsd.edu/. This iteration runs from 2013 to 2019 with 1/6° longitudinal grid spacing^34^. The meridional spacing varies with latitude such that Δx = Δy in meters. There are 52 vertical thickness-varying levels with 33 levels in the upper 750 m. Bathymetry is derived from the Earth TOPOgraphy 2 (ETOPO2) model^35^. The biogeochemical model is the Nitrogen version of the Biogeochemistry with Light, Iron, Nutrients, and Gas (N-BLING), which is evolved from the model by Galbraith et al.^36^. The B-SOSE model is of intermediate complexity, with nine prognostic tracers: dissolved inorganic carbon, alkalinity, dissolved oxygen, nitrate, phosphate, dissolved inorganic iron, dissolved organic nitrogen, dissolved organic phosphorus, and phytoplankton biomass. These gridded data products are easier to analyze and interpret than the profile data from the biogeochemical-Argo floats.

## Satellite data

South of 60°S satellites can only measure chlorophyll from ocean color sensors between October and March when there is sufficient light. To help validate the B-SOSE predictions, we compared the climatological values for February, averaged over 2013–2019, from the model-generated chlorophyll output with the chlorophyll data from the Visible Infrared Imaging Radiometer Suite (VIIRS) sensor on the Suomi National Polar-orbiting Partnership (SNPP) satellite. This period of comparison matches the time period of the B-SOSE model run used to estimate chlorophyll levels during winter months.

## Results

### Migration patterns

**Fig. 1:**
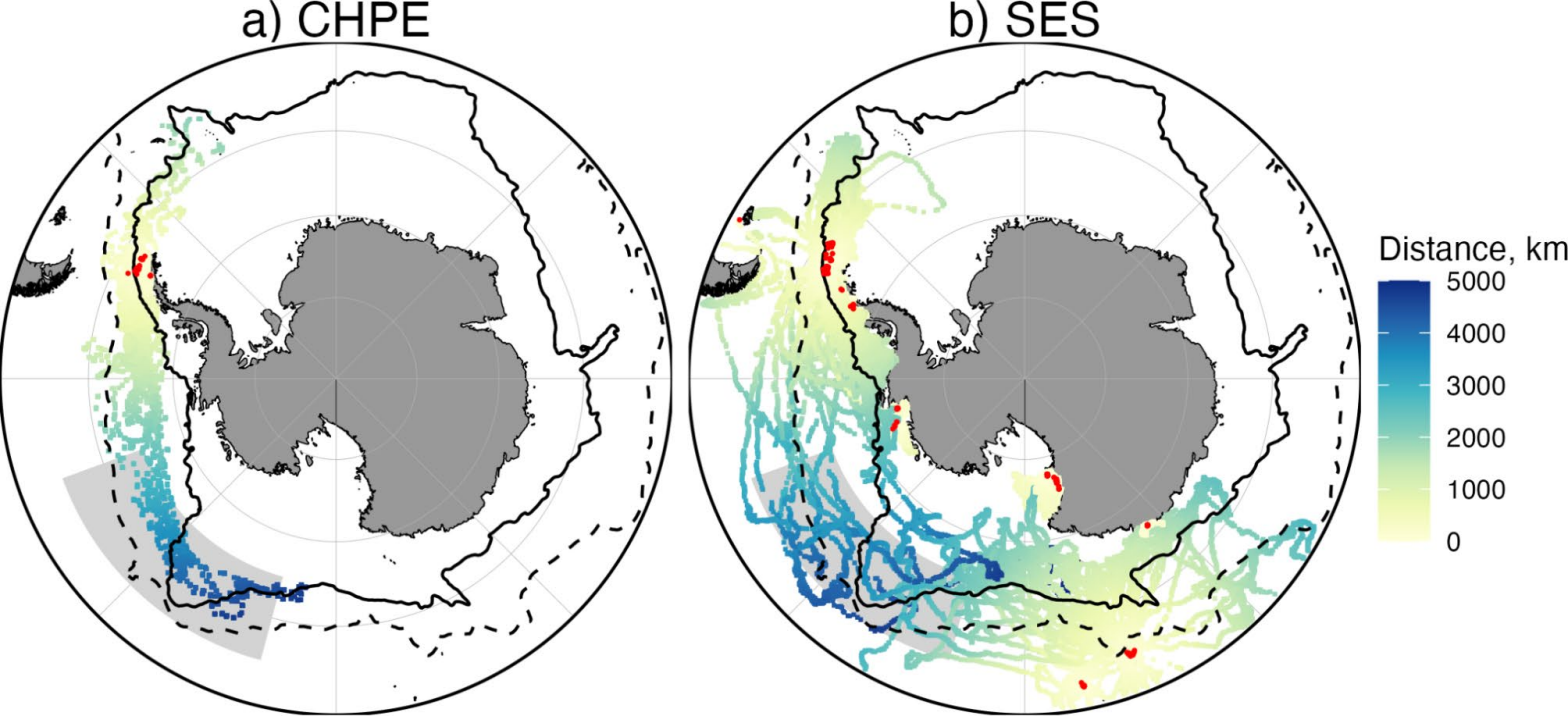
Maps showing the telemetry tracks for (**A**) chinstrap penguins (*N* = 74) and (**B**) southern elephant seals (*N* = 315), colored by the distance from the tagging location. The tagging locations are shown in red. The Polar Front (dashed line) and the southern boundary of the Antarctic Circumpolar Current (solid line), as provided by Orsi et al. (1995), are plotted for reference.The light gray box denotes the focus area between 55°S and 65°S and 110°W and 165°W. Maps were generated with R software, version 4.1.1.

**Fig. 2:**
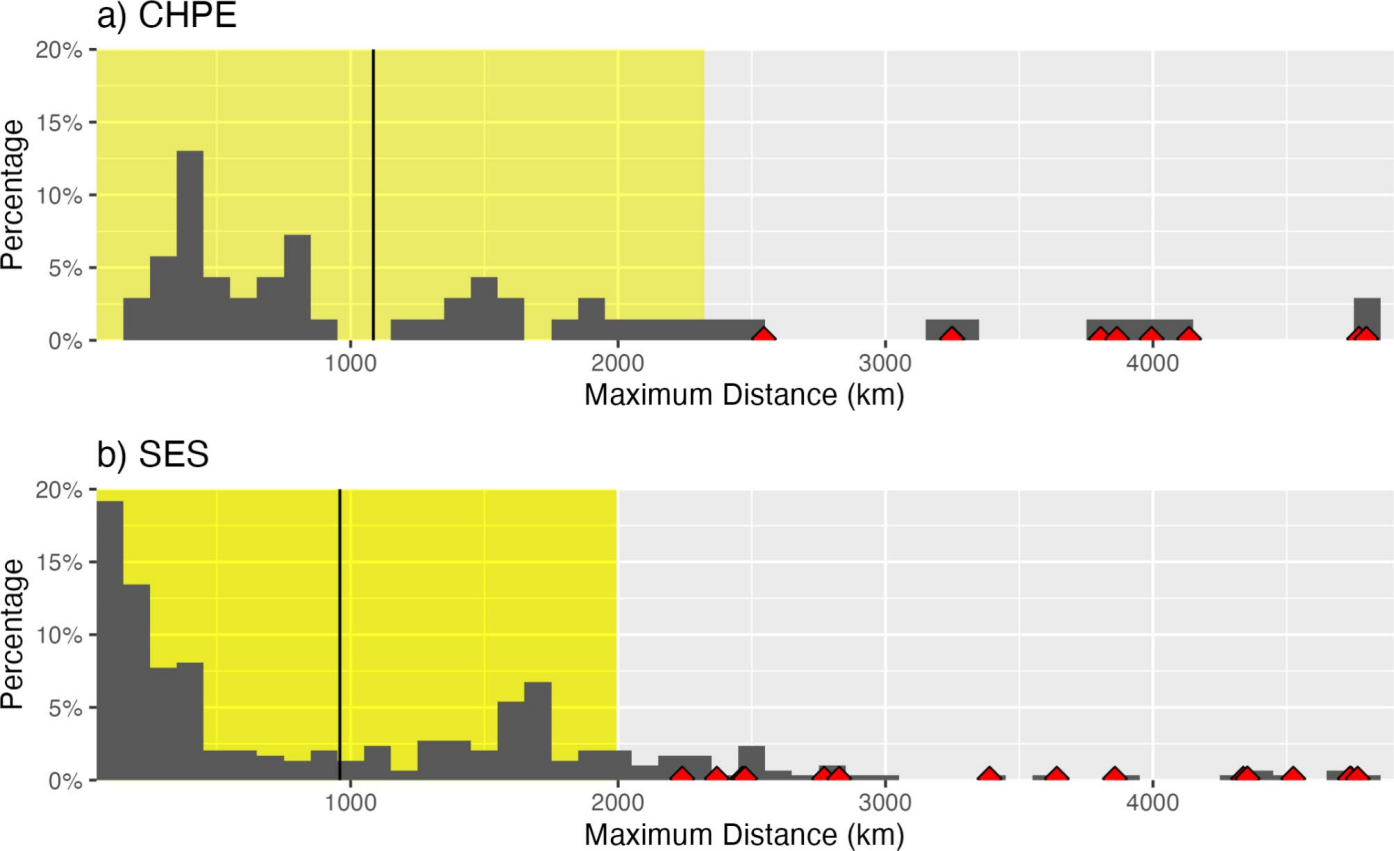
Histograms of maximum distance traveled from tagging locations for each tagged (**A**) chinstrap penguins and (**B**) southern elephant seals. The mean maximum distance for all individuals that did not migrate to the 140°W region is indicated by the black lines. The yellow shading shows the standard deviation of the mean of the maximum distance. The red markers indicate individuals that migrated to the 140°W region.

The overwinter migrations of CHPE and SES (Fig. 1) are similar in several respects. Both species exhibited a mix of movement patterns, with a predominance of relatively short movements (< 500 km) near tagging locations, but with some individuals undertaking much longer migrations (Fig. 2). In general, the mean maximum distance from the initial tagging locations that were achieved by the CHPE (1085 ± 123 km) and SES (960 ± 1035 km) were similar (Fig. 2). Both species have some tracks that demonstrate much longer migrations, on the order of 4000 km, and these all move into the region between 110°−160°W (Fig. 1). The longest penguin migration, originating in the South Shetland Islands region (60.9°W), reached 171.7°W, 4797 km from its tagging location, while the longest SES migration, which also originated from the South Shetland Island region, was 4747 km (Table 1).

**Fig. 3:**
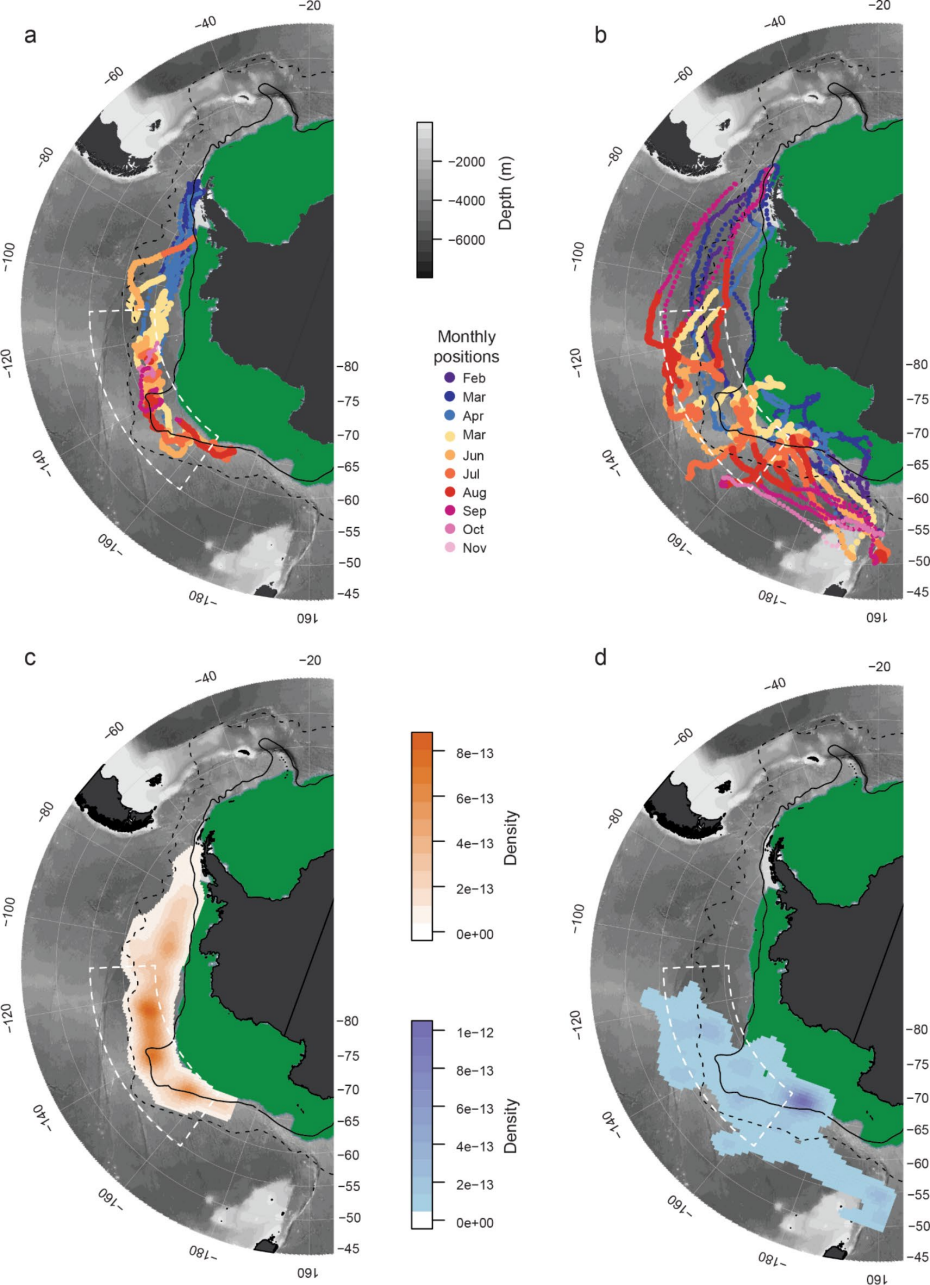
Location estimates, by month, for (**A**) chinstrap penguins (*N* = 9) and (**B**) southern elephant seals (*N* = 16) that exhibited movements into the 140°W region. Kernel density estimates indicating high-use areas for winter months (May-November) for **C**) chinstrap penguins and **D**) southern elephant seals. Bathymetry is from the ETOPO2 data set. Mean sea ice extent, in green, for June 2017 is plotted for reference. The Polar Front (dashed line) and the southern boundary of the Antarctic Circumpolar Current (solid line), as provided by Orsi et al. (1995), are plotted for reference. The dashed white lines denote the focus area between 55°S and 65°S and 110°W and 165°W. Maps were generated with R software, version 4.1.1.

Tracks for the subset of CHPE (*N* = 9) and SES (*N* = 16) that move into the study region are shown in Fig. 3. The CHPE generally stay between 60°−70°S in the southern portion of the ACC (Fig. 3a). This relatively narrow band of latitudes is driven by their habitat preference for sea surface temperatures < 2°C^11^. The SES tracks are distributed over a wider range of latitudes than the penguins, going both south and north of the ACC boundaries, with extensive use of the Ross Sea region (Fig. 3b). The SES that moved into the focus region originated from both the Antarctic Peninsula and the Macquarie/Campbell Islands (Fig. 1b, Table S1).

### KDE analysis

The kernel density analysis reveals distinct CHPE and SES hotspots in the Pacific sector of the Southern Ocean. There are two hotspots used by CHPE, one near 155°W and one between 120–140°W; both are between 60–65*°*S (Fig. 3c). Both hotspots occur along the southern boundary of the ACC. There are also two SES hotspots. The strongest occurs around 170°W, between 65–67°S, occurring slightly southwest of the CHPE hotspot at 155°W (Fig. 3d). This hotspot is centered slightly south of the southern boundary of the ACC. Another region with elevated SES density occurs between 120°−140°W, which overlaps with the larger CHPE hotspot (Fig. 3d). The hotspot near 140°W is located downstream of the narrow constriction in the ACC (Fig. 3c, d).

## Speed analysis

**Fig. 4:**
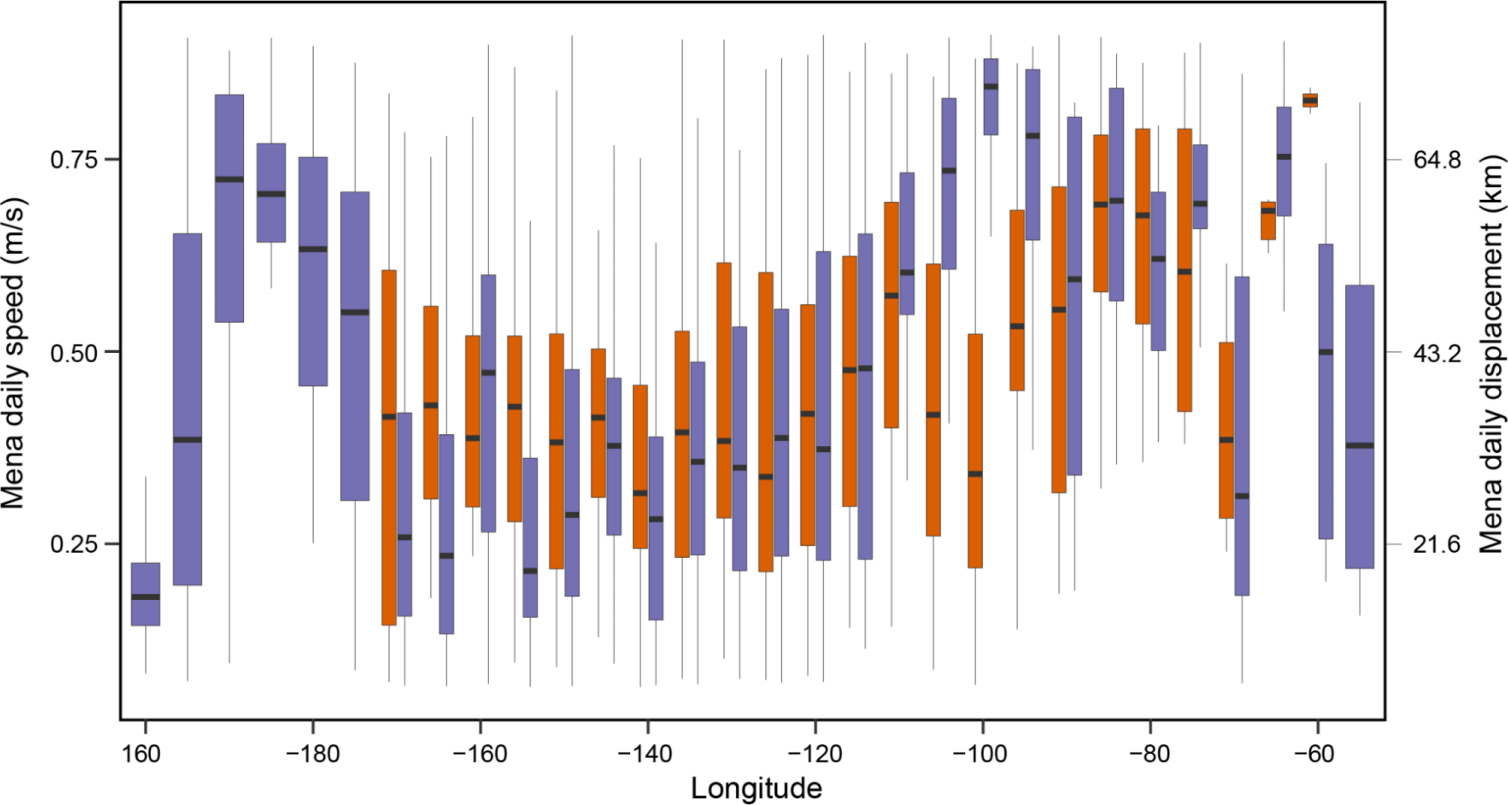
Boxplot of mean daily speed estimates within 5° longitude bins for chinstrap penguins (orange) and southern elephant seals (blue) that exhibited movements into the 140°W region. Median values are indicated by black dashes with the box and the range of data are represented by the whiskers.

Both species exhibited slower daily mean swim speeds between 120°−170°W relative to other longitudes (Fig. 4). For CHPE mean speeds slowed from 0.51 ± 0.30 m/s outside the region to 0.435 ± 0.229 m/s inside (t_425_ = 4.2, *p* < 0.01). For SES, mean speeds also slowed from 0.51 ± 0.38 m/s outside the region to 0.38 ± 0.31 m/s inside this region (t_2275_ = 8.9, *p* < 0.01). The reduced speeds equate with a reduction in daily displacement by 7 km for CHPE and 12 km, for SES. The relatively low swim speeds in the far east and west of the longitudinal range of the study region likely reflect local movements near breeding or molt locations.

## B-SOSE model output

**Fig. 5:**
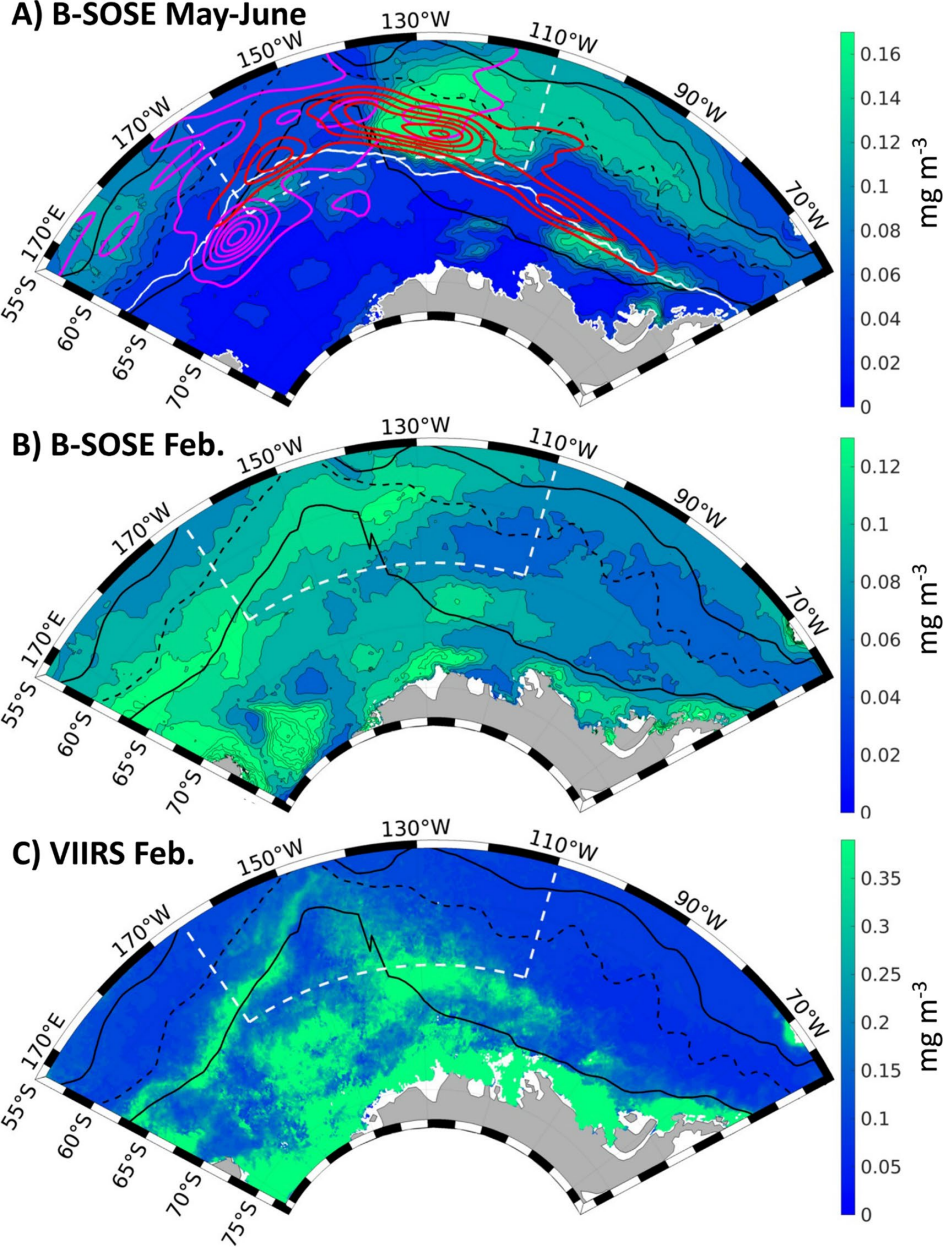
(A) Mean chlorophyll in May-June (austral winter) from the B-SOSE model. The solid white line denotes the May-June mean sea ice extent. The dashed white lines denote the focus area between 55°S and 65°S and 110°W and 165°W. The kernel density estimates shown in Fig. 3 are contoured here with 0.001 intervals in magenta for the southern elephant seals and red for the chinstrap penguins. (B) Mean chlorophyll concentration [mg m^−3^] in February (austral summer) from B-SOSE for the years 2013–2019, and (C) the equivalent from VIIRS. Model chlorophyll has a contour spacing of 0.02 mg m^−3^ in (A) and (B), but contours are omitted in the noisier satellite data for clarity. Similar to Fig. 1, black solid lines denote the Subantarctic Front and the southern boundary of the Antarctic Circumpolar Current, with the Polar Front shown as a black dashed line (Orsi et al., 1995). There is significant uncertainty in the magnitude, and the color bars are different in the panels. However, February chlorophyll in B-SOSE and VIIRS have similarities in the locations of greatest productivity, with both showing heightened chlorophyll along the southern boundary of the Antarctic Circumpolar Current. Maps were generated with Matlab software, version R2020b.

Output from the B-SOSE model shows the presence of enhanced chlorophyll year-round in this sector of the Southern Ocean (Fig. 5a, b). This large-scale chlorophyll distribution pattern is the same for all 7 years of the model output. The largest winter chlorophyll concentration, averaging over 0.17 mg/m^3^ (with a standard deviation of ~ 0.07 mg/m^3^), occurs between 115°−135°W, from the sea-ice edge into the ACC. There are also two smaller blooms. There is one with a magnitude of 0.13 mg/m^3^ (standard deviation of ~ 0.06 mg/m^3^) at the Polar Front and sea-ice edge in the Bellingshausen Sea (100°−80°W), and another with a magnitude ~ 0.10 mg/m^3^ (standard deviations of ~ 0.05 mg/m^3^) at the Polar Front and sea-ice edge of the central Ross Sea (165°−145°W). In comparison, outside of these bloom areas the background levels are chlorophyll are ~ 0.03 mg/m^3,^

The chlorophyll blooms observed in the B-SOSE model output can not be compared to satellite chlorophyll data since there is no satellite data south of 45°S from May to October due to insufficient light in winter. Climatological chlorophyll values for February from B-SOSE and the VIIRS satellite, however, exhibit similar qualitative patterns of elevated chlorophyll, although the magnitudes from B-SOSE are lower and the blooms smoother than the satellite data (Fig. 5b, c). Both show areas of elevated chlorophyll extending along the southern boundary of the ACC where the CHPE and SES hotspots occur, and also a notable bloom from about 140°W to 120°W where the CHPE hotspot occurs.

The hotspots for CHPE and SES overlap the areas of elevated chlorophyll. For the domain plotted in Fig. 5 (165°E to 60°W), the average chlorophyll in the area with a CHPE kernel density greater than 0.001 is 0.10 mg/m^3^, while the average with a lower kernel density is 0.08 mg/m^3^. CHPE tend to stay in a sea surface temperature range between 0 and 2 °C^11^, and if we only consider that range the average chlorophyll with a CHPE kernel density greater than 0.001 is 0.13 mg/m^3^, while the average with a lower kernel density is 0.09 mg/m^3^. For the SES, we only consider 165°E to 110°W as it is rare for the seals to travel further east. Here the average chlorophyll where the SES kernel density is greater than 0.01 is 0.08 mg/m^3^, while the average with a lower kernel density is 0.07 mg/m^3^.

## Discussion

We have identified two areas in the Pacific sector of the Southern Ocean that individual CHPE and SES appear to target during their long (> 4000 km) winter migrations. This behavior was observed in multiple years, in 4 of the 6 years of tag data for CHPE and in 7 of the 16 years of tag data for SES (Table 1). Animal behavior within the hotspots was characterized by slower average swim speeds, and may suggest occupancy of an area of increased importance for the predators relative to habitats with relatively faster migratory movements. The two hotspots occurred mainly between 60°and 65°S. One was centered north of the Ross Sea between 150°W and 170°W, where the CHPE and SES hotspots exhibited some spatial separation. The second was located between 120–140°W at the boundary between the Amundsen Sea and the Ross Gyre, and is primarily occupied by CHPE, though elevated use by SES relative to the surrounding regions is also apparent here. These hotspots of predator use overlap with regions predicted to have persistent and elevated chlorophyll during winter, indicating the importance of bottom-up processes to migratory marine predators in remote ocean regions.

These two areas of consistent use by CHPE and SES have not previously been identified as biological hotspots, despite a number of studies that have used tracking data to identify important habitats in the Southern Ocean. A study^38^of seal and penguin tracking data discovered areas of productivity created by seamounts in the Atlantic and Indian sectors of the Southern Ocean but did not examine any data from the Pacific sector^38^. Another analysis of tracking data from bird and mammal species in the Southern Ocean identified large Areas of Ecological Significance (AESs) in the Atlantic and Indian sector of the Southern Ocean, but none in the Pacific sector^14,37^. In that study, the species examined included SES, but not CHPE^37^. Importantly, that study identified globally important AES by averaging species-specific maps of habitat importance, tending to weight areas with high species diversity more. Our approach differs; we explored a specific region that was visited regularly by migrating CHPE and SES to understand plausible mechanisms that might promote the use of such remote pelagic regions. The identification of elevated marine productivity in the study region that overlaps areas of increased predator use warrants further attention. For example, Hinke et al.^11^ analyzed a single year of the data presented here and suggested that individual variability in migratory movements was a dominant driver for the broad-scale patterns of habitat use observed across the Southern Ocean. However, by extending that data set to include multiple years of tracking data from two species, we find evidence for a repeatable pattern of habitat use during winter that suggests the importance of the region. Such predictable migratory destinations represent important wintering habitats and provide an opportunity for future work to study the effects of variable marine productivity during winter on migratory animals and to link variation in migratory habitats with population trends for these important Antarctic predator species. Moreover, the region may represent important habitat for other species during winter, including emperor penguins (*Aptenodytes forsteri*)^38^ and crabeater seals (*Lobodon carcinophaga*)^14^. Despite these species’ general preference for pack-ice habitats during winter, prior tracking studies^14,38^ suggest that animals may occupy the areas of elevated winter chlorophyll identified here.

A summer chlorophyll bloom in this area has also been seen in previous analyses of satellite data^16,19,20^and, in part, is attributable to the turbulence resulting from the interaction of the ACC with the Pacific Antarctic Ridge. In particular, the UFZ around 144°W is a location of extreme topographical steering leading to heightened eddy activity downstream between 140°−135°W and 56°−58°S^17^. This physical mechanism for enhanced mixing supports the hypothesis that the elevated primary production near the boundary of the ACC, Amundsen Sea, and Ross Gyre promotes a productive pelagic habitat capable of supporting multiple predator species and their diverse foraging niches.

Output from the B-SOSE model provides evidence of an annual winter bloom within the eastern portion of our focus region area. The persistence of elevated chlorophyll may enhance local production of prey resources (krill, fish, squids), providing a productive and predictable habitat in this region. For example, while no commercial fisheries operate in this pelagic region currently, a small harvest of Antarctic krill prior to 1990 in the focus area^39^supports this assertion of a productive region for key prey resources. Moreover, the tracking data demonstrate movement into the bloom area, particularly for CHPE, in multiple years (4 of the 6 years of tag data). The CHPE hotspot between 120°–140°W overlaps with the area with the highest chlorophyll values in this sector. The model output also shows a region with elevated winter chlorophyll values (albeit less intense) that occurs along the southern ACC boundary, near 160°W. There, the CHPE hotspot occurs slightly north of the bloom, while the SES hotspot occurs west of the bloom. Since CHPE and SES target different prey^5,6,8^, and forage within different portions of the water column; it is possible that the spatial separation of their hotspots arises from spatial variability of prey resources associated with the area of elevated chlorophyll. For example, CHPE typically forage within the upper 50 m of the water column^40,41^, while SES can dive much deeper and exploit resources at depths in excess of 1000 m^42^. Alternatively, the spatial separation of the CHPE and SES winter hotspots north of the Ross Sea may be due to the presence of sea ice in the region. CHPE do not typically enter into the marginal ice zone (MIZ), favoring ice-free habitats^44^. The location of the bloom is likely to be partially covered by sea ice during mid-winter periods, forcing CHPE to remain further north relative to SES that can exploit the MIZ^43^. Regardless, the presence of consistently elevated local chlorophyll levels provides a bottom-up mechanism to attract and retain migratory predators in this remote habitat.

The SOCCOM project has generated a large amount of year-round, subsurface biogeochemical data for the remote Southern Ocean from the deployment of BGC-Argo floats. Our study was motivated by an interest in utilizing this novel data set in a project related to higher-trophic level predators and fishery management issues in the Southern Ocean, thus addressing a key priority of SOCCOM^44^. Despite the extensive number of SOCCOM floats deployed, however, the raw data remain too sparsely distributed for effectively overlaying them on the estimated locations from animal telemetry, as is standard practice when using gridded data products from remotely-sensed satellite observations. We therefore used the gridded fields of biogeochemical parameters from the output of the B-SOSE model which assimilates the BGC Argo float data. The year-round availability of BGC Argo float data in this remote part of the ocean is not only a valuable source of data for ocean ecosystem modeling, but we show that it is also useful for understanding the ecology of remote habitats used by migratory marine predators. Such ecological understanding can, in turn, help advance ocean management initiatives ranging from spatial protections like marine protected areas, to informing ecosystem-based fisheries management actions that are informed by the status and trends of non-target species. To maximize the use of these valuable BGC Argo float data for future ecological study and ecosystem management applications, we argue that it is important to continue efforts to make the float data available in gridded format, as either statistically mapped products, or as the output of models which assimilate the float data, as was used here. Similarly, efforts are needed to make these gridded products easily discoverable and accessible, specifically by providing means to both visualize the data and subset the data when downloading it. Issues of data accessibility are especially important to consider as the array of floats expands with the Global Ocean Biogeochemistry Array (GO-BGC)^45^.

## Conclusions

Roughly 10% of the CHPE and SES populations tracked over the last 3 decades have undertaken long-distance winter migrations, often traveling > 4000 km, to a remote region in the Pacific Sector of the Southern Ocean. We identified two hotspots of increased use by CHPE and SES that corresponded to broader regions of elevated chlorophyll predicted by the B-SOSE model. By leveraging in situ observations of biological variables during winter periods that derive from BGC Argo floats, we have shed light on a large-scale phenomenon invisible to traditional satellite sensors and linked long-distance migration patterns of two Antarctic marine predators with areas of consistently elevated chlorophyll levels. While the hotspots in the region are known areas of enhanced mixing^15–17^, this is the first study to show that it is likely a productive habitat capable of supporting diverse foraging niches exemplified by southern elephant seals and chinstrap penguins.

## Electronic supplementary material

Below is the link to the electronic supplementary material.

Supplementary Material 1

## Data Availability

The chinstrap penguin telemetry data for 2000, 2004, 2006, 2010, and 2011 are available from the Seabird Tracking Database at http://seabirdtracking.org. Chinstrap penguin telemetry data from 2017 are available at https://doi.org/10.1371/journal.pone.0226207.s008. The elephant seal telemetry data were obtained from two sources: the “Marine Mammals Exploring the Oceans Pole to Pole” (MEOP) consortium website at https://www.meop.net/ and the Tagging of Pelagic Predators dataset at https://coastwatch.pfeg.noaa.gov/erddap/tabledap/gtoppAT.html. Output from the B-SOSE model is available at http://sose.ucsd.edu/SO6/ITER135/. The VIIRS/SNPP data were obtained from the NASA Goddard Space Flight Center, Ocean Ecology Laboratory, Ocean Biology Processing Group. Visible and Infrared Imager/Radiometer Suite (VIIRS) Chlorophyll Data; NASA OB.DAAC, Greenbelt, MD, USA. doi:10.5067/SUOMI-NPP/VIIRS/L3M/CHL/2022. Accessed on 4 March 2020.
