# Supplementary material for: Long-distance winter migrations of chinstrap penguins and elephant seals to a persistent bloom at the edge of the Ross Gyre
Source: Sci Rep. 2025 Mar 21;15:9867. doi: 10.1038/s41598-025-87433-6 (PMC11928577; doi:10.1038/s41598-025-87433-6)
Supplement: Supplementary file 1 — Supplementary Material 1 [file 41598_2025_87433_MOESM1_ESM.docx]

**Supplemental Table S1**.Summary of tags for chinstrap penguins (CHPE) and southern elephant seals (SES) used in the analysis including deployment year, the number of days spanning all reported location estimates, the interval of reporting frequency, the hours of reporting , and the mean ± 1 SD of the number of location estimates available for each reporting period, and the maximum distance achieved from the original tagging location. Track origins are the South Shetland Islands (SSI), Falkland Islands/Malvinas (FI/M), Macquarie Island (MAC), or Campbell Island (CI).

| Species | ID code | Project | Origin | Year | N days | Interval | Hours of reporting | N daily locations | Max distance (km) |
| --- | --- | --- | --- | --- | --- | --- | --- | --- | --- |
| CHPE | 52505 | US-AMLR | SSI | 2006 | 122 | 3-day | 0000-1200 | 7.27 ± 3.95 | 2536 |
| CHPE | 98499 | US-AMLR | SSI | 2010 | 142 | 3-day | 0000-1200 | 15.83 ± 5.43 | 3851 |
| CHPE | 102171 | US-AMLR | SSI | 2011 | 163 | 3-day | 0000-1200 | 14.61 ± 5.37 | 3238 |
| CHPE | 102175 | US-AMLR | SSI | 2011 | 110 | 3-day | 0000-1200 | 9.13 ± 4.13 | 3790 |
| CHPE | 102176 | US-AMLR | SSI | 2011 | 94 | 3-day | 0000-1200 | 9.47 ± 3.98 | 3980 |
| CHPE | 102178 | US-AMLR | SSI | 2011 | 189 | 3-day | 0000-1200 | 11.6 ± 5.46 | 4753 |
| CHPE | 102179 | US-AMLR | SSI | 2011 | 100 | 3-day | 0000-1200 | 12.97 ± 3.87 | 3236 |
| CHPE | 165154 | US-AMLR | SSI | 2017 | 254 | Daily | 1200-1800 | 8.08 ± 3.24 | 4120 |
| CHPE | 165212 | US-AMLR | SSI | 2017 | 187 | Daily | 1200-1800 | 7.64 ± 3.17 | 4779 |
| SES | ct6-10011-05 | MEOP | MAC | 2005 | 213 | Daily | All day | 2.15 ± 1.03 | 2467 |
| SES | ct9-28587-05 | MEOP | SSI | 2005 | 216 | Daily | All day | 2.38 ± 0.97 | 4724 |
| SES | ct14-111-06 | MEOP | SSI | 2006 | 102 | Daily | All day | 2.29 ± 0.88 | 4507 |
| SES | ct37-590-08 | MEOP | SSI | 2008 | 225 | Daily | All day | 2.77 ± 0.97 | 4323 |
| SES | ct48-060-09 | MEOP | SSI | 2009 | 144 | Daily | All day | 2.66 ± 1.11 | 4718 |
| SES | ct56-M981-10 | MEOP | SSI | 2010 | 210 | Daily | All day | 2.94 ± 0.94 | 3843 |
| SES | ct56-R021-10 | MEOP | SSI | 2010 | 208 | Daily | All day | 2.14 ± 1.12 | 4336 |
| SES | ct64-M037-09 | MEOP | MAC | 2010 | 243 | Daily | All day | 3.36 ± 0.7 | 2818 |
| SES | ct64-M721-09 | MEOP | MAC | 2010 | 228 | Daily | All day | 3.41 ± 0.76 | 2361 |
| SES | ct64-M746-09 | MEOP | MAC | 2010 | 266 | Daily | All day | 3.32 ± 0.74 | 3377 |
| SES | ct64-M752-09 | MEOP | MAC | 2010 | 271 | Daily | All day | 3.31 ± 0.73 | 2457 |
| SES | ct64-M994-09 | MEOP | MAC | 2010 | 248 | Daily | All day | 3.3 ± 0.75 | 3628 |
| SES | ct79-238-12 | MEOP | CI | 2012 | 285 | Daily | All day | 3.25 ± 0.8 | 2232 |
| SES | ct100-301-13 | MEOP | CI | 2013 | 121 | Daily | All day | 3.37 ± 0.74 | 2761 |
| SES | 7608001 | TOPP | SSI | 2008 | 243 | Daily | All day | 3.65 ± 2.12 | 4339 |
| SES | 7609001 | TOPP | SSI | 2009 | 126 | Daily | All day | 4.54 ± 2.64 | 4747 |
